# Consensus statement: osteoporosis prevention and treatment in Latin America—current structure and future directions

**DOI:** 10.1007/s11657-018-0505-x

**Published:** 2018-08-24

**Authors:** Ben-Hur Albergaria, Monique Chalem, Patricia Clark, Osvaldo Daniel Messina, Rosa Maria R. Pereira, Luis F. Vidal

**Affiliations:** 1 Diagnosis and Research Center of Osteoporosis (CEDOES), Universidade Federal do Espírito Santo (UFES), Vitória, ES Brazil; 2 Fundación Santa Fe de Bogotá, Bogotá, Colombia; 3 Clinical Epidemiology Unit, Hospital Infantil Federico Gómez, Mexico, D.F. Mexico; 4 Faculty of Medicine, Universidad Nacional Autonoma De Mexico, Mexico, D.F. Mexico; 5 Rheumatology Service, Cosme Argerich Hospital and IRO Clinical Research Center, Buenos Aires, Argentina; 6 Rheumatology Division, Hospital das Clinicas HCFMUSP, Faculdade de Medicina, Universidade de Sao Paulo, Sao Paulo, SP Brazil; 7 Rheumatology Service, Centro Diagnóstico de la Osteoporosis y Enfermedades Reumáticas (CEDOR), Lima, Peru

**Keywords:** Latin America, Burden of osteoporosis, Osteoporosis diagnosis and treatment, Health care in Latin America

## Abstract

**Background:**

Osteoporosis is a common disorder affecting populations worldwide. In Latin America, an aging population combined with limited health care resources result in osteoporosis quickly becoming a condition of considerable magnitude with disproportionate morbidity and mortality.

**Aim:**

To review the current state of prevention, diagnosis, and treatment of osteoporosis in Latin America and to develop strategies and recommendations that may be adopted in the region, an expert panel of clinicians and scientists was assembled to develop a consensus statement outlining future directions.

**Method:**

The panel conducted a comprehensive literature review of publications mainly related to osteoporosis in Latin America, and at an in-person meeting developed a consensus position to address the relevant issues.

**Results:**

The epidemiology, burden, diagnosis, and treatment of osteoporosis in the region were discussed with particular attention to issues unique to the region. A series of recommendations were developed encompassing virtually all aspects of the disease, including improved public and health professional awareness, better diagnostic processes, improved access to care, and greater engagement by health policy makers, government, and a wide variety of private organizations.

**Conclusions:**

The panel concluded that a comprehensive approach to osteoporosis prevention and treatment in Latin America is urgently needed.

Osteoporosis imposes a very high burden on both individuals and health systems worldwide. Osteoporosis is a systemic skeletal disease that is characterized by low bone mass and micro-architectural deterioration of bone tissue, with a consequent increase in bone fragility and susceptibility to fracture [1]. Fragility fractures, especially hip fractures, are the most serious consequence of osteoporosis, imposing a heightened risk for loss of independence, chronic pain, the need for rehabilitation, and excess mortality [2]. Moreover, fragility fractures can result in significant social, economic, and adverse effects due to prolonged hospitalizations, long-term nursing home care, need for surgery, medical treatments, and limitations in daily living.

Osteoporotic-related fractures are a major global epidemic. The number of fractures worldwide was about 9 million annually in 2000 [3]. The wordwide economic cost of osteoporosis is expected to rise to about $132 billion by 2050 [4]. Importantly, a study conducted by the Mayo Clinic [5] in women aged 55 years and over showed osteoporotic fractures accounted for more than 40% of the hospital admissions among osteoporotic fractures, myocardial infarction, stroke, and breast cancer. The cost of hospitalization was greater for osteoporotic fractures (US$ 5.1 billion) than for myocardial infarction (US$ 4.3 billion), stroke (US$ 3 billion), or breast cancer (US$ 0.5 billion) [5]. These data highlight the significant burden of osteoporosis and suggest that the prevention of fractures in high-risk individuals may be very impactful, particularly because fractures beget fractures so early treatment in at-risk patients is essential to preventing future fractures.

It is important to note that with aging societies and changing disease patterns worldwide, the burden of osteoporosis will continue to rise globally and likely more so in the Latin American region. Indeed, whereas 13–20% of the population in the region is currently over 50 years of age, that proportion is estimated to increase to 28–40% in 2050 [6]. This demographic change may result in a disproportionate increase in hip fractures compared to other regions worldwide [7].

Despite these sobering facts, including the substantial impact of osteoporosis in terms of economic cost, morbidity, and mortality, the lack of recognition of osteoporosis and its under-treatment has been well documented [8]. In this paper, we briefly review the epidemiology, diagnosis, and treatment of osteoporosis, with particular attention to issues in Latin America (LA) and the current inertia in care. We then focus our attention on strategies and recommendations that may be adopted within the region, particularly seeking to increase the prioritization of osteoporosis as a health care priority and increase its early prevention, diagnosis, and treatment.

## Methods

To address the above issues, the AHF identified a six-member panel of clinicians and scientists from Latin America with expertise in the field of osteoporosis and representing the disciplines of rheumatology and gynecology. Both AHF and the expert panel independently used PubMed, Embase, Lilacs (Latin American and Caribbean Literature on Health Sciences), and Scielo (Scientific Electronic Library Online) to conduct a careful review of the literature. Useful publications were identified using terms related to osteoporosis, osteopenia, osteoporosis diagnosis, osteoporosis epidemiology, and, in PubMed, combining them with country names or regional names related to Latin American and the Caribbean Islands.

The search strategy for databases was (osteoporosis diagnosis) AND ((Latin America OR Central America OR South America OR Caribbean OR Argentina OR Bolivia OR Brazil OR Chile OR Colombia OR Costa Rica OR Cuba OR Dominican Republic OR Ecuador OR El Salvador OR Guatemala OR Haiti OR Honduras OR Mexico OR Nicaragua OR Panama OR Paraguay OR Peru OR Uruguay OR Venezuela))). A total of 1039 articles were identified through the online search: 842 in PubMed, 82 in Lilacs, and 115 in Scielo. Some of them were excluded as duplicates and most were excluded after screening title. The literature review was not directed toward identifying papers related to specific treatments, but rather focused on papers that reviewed or summarized activities related to osteoporosis in Latin America. In total, 48 relevant publications were shared among the entire panel prior to their in-person meeting.

To better focus the discussion, AHF staff developed a set of generic questions related to the topic, and distributed them to the panel members before the meeting. Panel members independently drafted a response to the questions, which served as the starting point for discussion at the meeting. During the course of a multi-day meeting, the panel reviewed and discussed the published literature, and from numerous drafts and rounds of discussion, a manuscript was created based on unanimous consensus. Subsequent to the meeting, the panel reviewed the document, minor edits were made, and the panel acknowledged that they were in full agreement.

## Epidemiology and burden

Most reports on the epidemiology of osteoporosis in LA come from 14 countries whose National Societies have membership to the International Osteoporosis Foundation. Additional studies gathered by the literature search or local publications are included as well. The reasons for a lack of data from other countries are likely to be the absence of national databases and robust surveillance methodology, reporting issues, and geographical and cultural barriers. What data is available can be grouped into the epidemiology of hip, vertebral, and other fractures.

The epidemiology in the region was summarized in the Latin America Regional Audit in 2012 [9]. Hip fracture rates in women age 50 and older ranged from 53 to 443 per 100,000 persons, and from 27 to 135 per 100,000 for men age 50 years and older, with a ratio of 2 to 3 women per man [9]. The lifetime risk of hip fracture after 50 years of age in Mexico was 8.5% in women and 3.8% in men. In Venezuela, the risk was 5.5% for women and 1.5% for men [10].

The projections of hip fractures in the region show that there will be a significant increase by the year 2050. It is estimated that in Mexico the annual number of hip fractures will increase from 29,732 in 2005 to 155,874 in 2050 [11]. In Argentina there are approximately 34,000 annual hip fractures among people over 50 years of age, with an average of 90 fractures per day [12]. It is estimated that the number of hip fractures per year in this population will almost triple by 2050. In Brazil, there are approximately 121,700 hip fractures per year, which are projected to increase to 160,000 annual fractures by 2050 [13, 14].

In Latin America the direct costs for hip fracture care vary from $3100 USD in Uruguay to $12,000 USD in Brazil [9]. In a study from Mexico, it was reported that in 2006, a little more than 97 million dollars was spent for hospital care for hip fractures. This expense was equivalent to the cost of insulin for the entire country’s insulin-dependent population for the same year [13, 15]. Another study in Mexico found that the annual cost of non-pharmacological management of osteopenia and osteoporosis, in addition to the medical care of the four main fragility fractures (hip, spine, forearm, and humerus) caused by osteopenia and osteoporosis, was 480 million of dollars in 2010. The projections for 2020 indicated that these costs will increase by 42% [16].

Vertebral fractures are the most common single osteoporotic fractures worldwide, and occur in 30–50% of people over age of 50 [17]. However, in contrast to hip fractures, 2/3 to 3/4 of these are clinically silent and less than 10% require hospital admission [18]. Moreover, even when there is a vertebral fracture on X-ray, it is often not recorded by the radiologist, rarely noted by the clinician, and consequently no treatment is provided.

Asymptomatic vertebral fractures are associated with decreased quality of life, regardless of age, body mass index, and physical activity, highlighting the importance of preventing these fractures [19]. The incidence rates of these fractures rise exponentially with age.

In 2009, the first multicenter study of radiographically identified vertebral fractures in the region was reported in a random sample of populations from Argentina, Brazil, Colombia, Puerto Rico, Mexico, and Venezuela. The study consisted of women over the age of 50 [13]. The data confirmed an exponential increase in fragility with age. The highest overall rate was in Mexico (19%) and the lowest in Puerto Rico (12%). Another study conducted in Brazil with 943 subjects older than 65 years (São Paulo Aging & Heath Study) found an overall prevalence of vertebral fractures of 29% (95% CI 26–32), with no difference between men and women [20].

Information related to other fragility fractures is scarce and comes primarily from Brazil and Mexico. The Brazilian study on osteoporosis (ARMS) [14], completed in 2009, showed that the prevalence rate of low-impact fractures ranged between 11 and 22% in men, and between 11 and 17% in women. In the Brazilian São Paulo Aging & Heath Study [21], the main fractures were distal forearm (6%), humerus (2%), and ribs (1%). In this study, a significantly higher prevalence was observed in women (17%) than in men (7%). Mexico has reported that nationwide, distal forearm fractures are the highest reported followed by hip and humerus fractures [22].

A fracture at any location results in an increased risk of subsequent fractures, with the highest risk following hip and vertebral fractures in men and women [23]. The subsequent fracture risk is not constant over time; patients are at highest risk immediately after the initial fracture is incurred, particularly in the first year. Data suggest that clinical vertebral and non-vertebral fractures cluster in time from menopause onwards. Up to 23% of the subsequent fractures occur within 1 year after the first fracture, and about 54% occur within 5 years [24]. The risk of fracture decreases with time, but does not return to baseline values [25]. In spite of the high rate of refractures, treatment with anti-resorptive drugs following a fracture is very low, ranging from 5 to 44% of eligible patients [26].

The majority of patients with any fragility fracture are not treated to prevent another fracture, even when effective treatments are available. A recent prospective observational study of > 60,000 women aged ≥ 55 years, recruited from 723 primary physician practices in 10 countries, reported that less than 20% of women with new fractures received an osteoporosis diagnosis and treatment [27]. There is a paucity of data about this situation among LA countries, but, at least in the Brazilian experience, the majority of patients with fractures due to fragility are neither evaluated nor treated for osteoporosis or for the prevention of subsequent falls [28].

## Diagnosis and treatment in Latin America

The World Health Organization first convened a group of experts in 1994 to assess fracture risk and its application to screening for post-menopausal osteoporosis. Osteoporosis was defined based on bone mineral density. A standardized score, called T-score, was used to define the categories for diagnosis, which are as follows:

- Normal (T-score − 1.0 and above)
- Low bone mass, referred to as osteopenia (T-score between − 1.0 and < − 2.5)
- Osteoporosis (T-score − 2.5 and below)
- Severe osteoporosis (T-score − 2.5 and below with history of a fracture).

A dual X-ray absorptiometry (DXA) assessment will determine bone mineral density. The assessment by DXA is the gold standard for the diagnosis of osteoporosis and is an important component of assessing fracture risk. However, given the high specificity but low sensitivity of using bone mineral density for risk prediction, the combination of DXA and clinical risk factors, such as FRAX, significantly improves the sensitivity of risk prediction [29]. Moreover, the use of FRAX thresholds can reduce the need for DXA in patients identified as having either a high or low risk of osteoporotic fracture, and in some cases, patients at high risk may be considered for treatment even without BMD testing [30]. DXA is recommended for women aged 65 and older, in post-menopausal women less than 65 with risk factors, or in men age 70 and older and in younger men if they have risk factors for low bone mass [31].

One valuable tool to assess fracture risk and assist in treatment decisions is a risk calculator. The osteoporosis field has developed validated fracture risk calculators [30] that also delineate intervention thresholds for osteoporosis drug therapy. In this paper we focus on the FRAX risk calculator because of its widespread availability and use.

FRAX uses several osteoporosis risk factors and provides a 10-year probability of hip fracture and a 10-year probability of a major osteoporotic fracture (i.e., clinical vertebral fracture, spine, wrist, hip, or shoulder fracture). The FRAX index has been validated in several LA countries (Argentina, Brazil, Chile, Colombia, Ecuador, Mexico, and Venezuela), and more recently, FRAX-based therapeutic thresholds were published [32]. A FRAX assessment should be made in all men over 50 and in all post-menopausal women with a clinical risk factor or a BMI < 19 kg/m^2^ [30]. In older populations (i.e., women over 65 and men over 75), age itself is a strong enough risk factor to warrant risk assessment. Different countries have different thresholds for recommended treatment according to FRAX values.

It is important to identify those patients who need screening, diagnosis, and treatment as soon as possible. In LA countries, a FRAX-based intervention threshold identifies those who may warrant treatment. The heterogeneity of the intervention threshold between LA countries indicates that country-specific FRAX models are more appropriate than would be a single FRAX model [32].

The advantage of FRAX compared to DXA is that the former is easily available, inexpensive, the test can be performed in a wide variety of locations, and requires less health professional training to administer. It is an ideal method of screening in primary care. It is also particularly useful in borderline cases of osteopenia, in which a patient may have a high FRAX risk, but does not yet have a T-score below − 2.5 [32].

Treatment should begin if there is a previous fragility fracture or if the T-score (i.e., DXA) is below or equal to − 2.5 at lumbar spine, total hip or femoral neck, or who are osteopenic and have a FRAX score above the designated interventional level. The drugs available either decrease bone resorption (anti-resorptives) or are anabolic agents (stimulate bone formation). Anti-resorptive therapies include bisphosphonates, denosumab, hormone replacement therapy, and selective estrogen receptor modulators (SERMs). The only bone forming therapy currently available in Latin America is teriparatide.

In addition to pharmacologic therapy, all patients should receive lifestyle modification recommendations (e.g., recommendations for physical activity, smoking cessation, nutrition counseling, and alcohol intake of < 3 units per day) and adequate calcium consumption by diet or supplements, and appropriate vitamin D supplements. Region-wide guidelines for diagnosis and treatment of osteoporosis have not been established; however, many countries have their own guidelines that specify treatments as first- or second-line options and the duration of treatment, among other relevant to that country.

Compliance with therapy is a complex process that is influenced by multiple factors. A lack of adherence to therapies is an issue for most chronic diseases, including osteoporosis. In the real world, adherence to treatment is much less than in controlled clinical trials. A lack of adherence to osteoporosis treatment will ultimately have an effect on the degree of fracture protection achieved [33]. There appear to be no studies in LA that evaluate adherence to therapy.

## Issues related to Latin America

Since 1990, every country in the region has gone through a series of health sector reforms with the aim of increasing equity, effectiveness, and coverage. Unfortunately, despite some positive results, these reforms have not achieved their anticipated goals. The mixed composition of health systems, social and health determinants, and varying economic conditions are important issues related to access to quality health care in the region. While some health care systems provide DXA and access to certain types of drugs, others do not. As a result, there is no universal access to prevention interventions or treatment options across the region.

LA health systems are highly fragmented [34] and there is a lack of coordination between systems in a country [35]. Another important factor in LA is that approximately 21% of the population does not seek care because of geographical barriers [9].

Although pharmacologic agents to prevent and treat osteoporosis are widely available in LA, some unique disparities exist. In most countries there is at least a public health care system and a self-funded private insurance system. Both systems provide widely available, inexpensive, and effective agents (e.g., bisphosphonates), but other drugs (e.g., denosumab, teriparatide) are sometimes only available through the private insurance system. Thus, low-income patients often cannot attain access to medications that may be medically indicated.

All told, osteoporosis is not a priority for public health in most LA countries, where basic health problems such as infant mortality, vaccination, and other health problems consume most of the national budget for health. Other chronic diseases with high costs, such as cancer and diabetes, also account for a high percentage of the budget. Given the enormous toll that osteoporosis imparts on populations, and the relatively low cost of effective treatments, it is clear that much more needs to be done to elevate the commitment by the public, health professionals, and policy makers to reduce the adverse outcomes associated with this disease.

## Future directions

### Epidemiology

An important action in the future would an emphasis on greater data collection related to osteoporosis and osteoporotic fractures, as well as the design of appropriate epidemiological studies using a methodology that can be shared by different countries to study the health determinants and impact of osteoporosis. Some countries in the region have studies on the cost of fractures, but most do not. One action would be to promote both data collection of fracture incidence, and studies of fracture costs in those LA countries that lack this information.

### Guidelines

The value of evidence-based guidelines for osteoporosis management is widely recognized. Health authorities should disseminate the guidelines and audit changes in practice derived from their application [36]. Medical societies and other professionals of each country should also be encouraged to develop or update management guides, improve the availability of DXA equipment and other diagnostic resources, identify local risk factors, and enhance the availability of medicines in each country.

### Diagnosis and treatment tools

Another important strategy is to have diagnostic technology available throughout LA, including in small towns. Unfortunately, there is a clear trend to concentrate technology in big cities. As mentioned above, FRAX is an important tool to assess risk and guide treatment, and its dissemination would be invaluable. In Mexico, FRAX was simplified and adapted as a paper test, since there is sparse Internet availability in primary care facilities. Health providers are being trained in osteoporosis and in the use of FRAX through frequent webinars and courses, and FRAX is now being tested in about 10,000 primary care facilities in that country [37].

The need for widespread availability of DXA equipment is also critical since this methodology provides the most accurate assessment of fracture risk and the effectiveness of treatment. The inclusion of the DXA exam has been an integral strategy in the guidelines for osteoporosis detection and management. In some LA countries, however, there are few DXA machines and accessibility in rural areas is difficult. Inequities can result in long waiting times or long distances to travel or, in many cases, no practical access to DXA at all [9]. This situation generates an imbalance because the majority of the population has access to public hospitals where the resource is limited and the wait time is long [9]. Much work remains to make DXA machines more widely available in LA.

#### Awareness

Osteoporosis remains a relatively undetected and untreated disease. Awareness, prevention, and diagnosis of osteoporosis are paramount to contain the social and financial consequences of this disease in LA. The accomplishment of these tasks, however, faces unique challenges. Much of the population in LA has little access to medicine and great disparities exist in access to the public health care system, with widespread difficulty to obtain specialized medical attention.

At the governmental level, there are currently no national public awareness programs on osteoporosis; osteoporosis is a national health priority in only three countries in the region—Brazil, Cuba, and Mexico [9]. Thus, increasing the visibility of osteoporosis is very important, and should be targeted to (a) the general public, (b) health providers, and (c) health authorities.

### General public

The self-perception of osteoporosis and fracture risk in Latin American women has received some attention. Data from one study from Brazil showed that post-menopausal women significantly underestimate their risk of osteoporotic fractures; almost 80% of women identified as having a high risk of fractures by FRAX perceived themselves as having little risk [38].

Proper nutrition, physical activity, a healthy lifestyle (smoking cessation, heavy alcohol avoidance), and the prevention of falls are the hallmarks for osteoporosis prevention. Public awareness campaigns that address these issues are greatly needed. For example, the “Best Bones Forever” program [39] is a US national campaign targeting young girls and teens (9 to 14 years old) to promote healthy habits for bone growth and fracture prevention. Local medical societies should evaluate the different campaigns to determine if any of them can be implemented in their community.

### Health providers

It is of the utmost importance to target those providers that deliver primary and secondary prevention so that they are fully aware of how to screen those at high risk and the actions necessary for secondary prevention. In the case of primary prevention, primary care physicians, family physicians, and gynecologists are the most important target groups, and in the case of secondary prevention, the orthopedic surgeon is the key professional. These physicians must become aware of the seriousness of osteoporosis, and be trained in how to detect osteoporosis.

Organizations such as the International Osteoporosis Foundation (IOF) and National Osteoporosis Foundation (NOF) have developed materials for patients on their web pages that can be used, or as examples of materials these can be adopted for populations in LA. Patient societies have helped increase public awareness in LA, but much more can be done. World Osteoporosis Day, every 20 October, is also a government platform to highlight new programs and disseminate key messages. Moreover, the entire region could benefit greatly from the establishment and strengthening of more and effective organizations that represent the interests of the public in osteoporosis prevention and treatment, including via the greater participation of patient advocates in national osteoporosis foundations.

### Health authorities

Health professionals and medical societies have the responsibility to impart knowledge to health authorities about osteoporosis and its consequences. Without the support of the government, barriers to effective health care will continue to exist. The funding and implementation of awareness campaigns is an essential government activity. Additional support should be solicited from the pharmaceutical and food industries.

#### Comprehensive programs

A good example of a multi-national plan to reduce the impact of osteoporosis has been developed and implemented by the European Community (EC). The EC plan includes public and relevant health professional awareness campaigns, the dissemination of preventive lifestyle measures, the development of evidence-based guidelines, recommendations for fracture care, rehabilitation and prevention of falls, and the development of economic models that demonstrate how treatment can be most effective [40]. The feasibility of applying this approach to LA should be carefully considered.

Another comprehensive approach to addressing the burden of osteoporosis has been implemented in Australia [41]. This program consists of many strategies that may also be appropriate for the region and its countries. Briefly, the elements of a region-wide program to prevent and treat osteoporosis could include the following:

1. A Region-Wide Steering Committee—The current Latin American advisory committee of the IOF should be strengthened. The activities of this committee could be expanded to help communicate and coordinate actions between countries, disseminate information regarding each country’s activities, and standardize measures across the region.
2. National Leadership Committees—These independent committees, funded by the government and/or pharmaceutical industry in each country, would facilitate the collection of data on the incidence and prevalence osteoporosis and its risk factors. Also, they would establish awareness campaigns in each country targeted toward health professionals and the public, and include a refracture prevention initiative to follow-up and coordinate the care of those who develop a fragility fracture. The National Leadership Committee might also address such important issues such as the following:

- How can hospital and community-based teams best work together to provide an integrated local fracture liaison service and where do accountabilities lie?
- How can data be better shared to support care coordination?
- How to connect with aged care facilities and services?
- How to improve services in remote and rural locations?
- What further barriers need to be considered and overcome?

### Performance measures

Another approach that has been used in many countries is to develop, implement, and track what are termed “performance measures.” Such measures exist in the USA and in some countries in Latin America for osteoporosis, and health care delivery institutions are required to report their success on these measures. In this fashion, medical delivery systems are made accountable for their ability to provide quality health care. Some examples of performance measures include the following:

A. The proportion of women 65–85 years of age who suffered a fracture received either a DXA measurement or a prescription for a drug to treat osteoporosis within 6 months of fracture.
B. The proportion of patients over 50 who receive a FRAX assessment.
C. The proportion of post-menopausal women with risk factors between ages 50 and 65 who receive a DXA measurement.

### Post-fracture care

Fracture care, rehabilitation, and prevention of falls are another important step and require the participation of a multidisciplinary team that should include at least the primary care physician, orthopedic surgeon, and a physician specializing in rehabilitation [36, 42, 43]. A fall prevention program for older adults is also a useful resource and could be organized and implemented by health personnel such as nurses or rehabilitation specialists.

The IOF and the American Society for Bone and Mineral Research published position papers on coordinator-based systems for secondary prevention in fragility fracture patients. Exemplar service models were referred to as “Fracture Liaison Services (FLS),” while other terms are used in different countries. These services facilitate education about osteoporosis, the assessment of bone mineral density, and the care of patients who have suffered a fragility fracture [44, 45]. Within the larger program, there is an effort titled “Capture the Fracture.” This program offers a framework to manage the risk of secondary fractures. Adapting the FLS model to the region could be an important way to reduce refracture rates and their associated costs.

## Summary

In conclusion, osteoporosis and its related fractures constitute a major public health in LA and there are projections of an exponential increase in osteoporotic fractures in the region over the next decades. These trends show the need for timely and effective diagnosis of osteoporosis to reduce the disease burden in LA. The low self-awareness of osteoporosis and fracture risk among LA post-menopausal women and their health care providers, and limited access and utilization of bone densitometry and FRAX, are all important issues for immediate resolution. Health policy makers, insurance providers (both public and private), medical societies, employer organizations, hospitals, long-term care facilities, patients, and the general public should work to break the treatment inertia in osteoporosis by reevaluating the importance of osteoporosis prevention, treatment, and management as a health care priority. LA must overcome all these issues in order to achieve the goal of preventing unnecessary fractures.
